# Adherence to recommendations of inpatient geriatric consultation teams: a multicenter observational study

**DOI:** 10.1007/s41999-020-00397-w

**Published:** 2020-09-25

**Authors:** Mieke Deschodt, Anthony Jeuris, Bastiaan Van Grootven, Eline Van Waerebeek, Evie Gantois, Johan Flamaing, Anja Velghe

**Affiliations:** 1 Department of Public Health and Primary Care, Gerontology and Geriatrics, KU Leuven, Leuven, Belgium; 2 Department of Public Health, Nursing Science, University of Basel, Basel, Switzerland; 3 Jessa Ziekenhuis, Hasselt, Belgium; 4 Department of Public Health and Primary Care, Academic Center for Nursing and Midwifery, KU Leuven, Leuven, Belgium; 5 Research Foundation Flanders (FWO), Brussels, Belgium; 6 Ghent, Belgium; 7 Geriatrics Department, University Hospitals Leuven, Leuven, Belgium; 8 Geriatrics Department, University Hospitals Ghent, Ghent, Belgium

**Keywords:** Geriatric assessment, Hospital, Referral and consultation, Treatment adherence and compliance

## Abstract

**Purpose:**

Inpatient geriatric consultation teams (IGCTs) provide recommendations for the care of older patients hospitalized on non-geriatric wards based on comprehensive geriatric assessment. The lack of adherence to their recommendations hinders the potential impact of IGCTs. We evaluated the adherence to IGCT recommendations and determined which team and recommendation characteristics are related to higher adherence rates.

**Methods:**

Multicenter observational study in older adults aged ≥ 75 years admitted to an acute non-geriatric ward. Demographic and adherence data were collected for 30 consecutive patients. A cross-sectional survey mapped team and organizational characteristics of the participating IGCTs.

**Results:**

Analyses were conducted in 278 patients (51.4% male, mean age 82.5 years, and median length of stay 10 days). There was a median number of 3 recommendations (range 1–13) per patient. The overall adherence rate was 69.7%. Recommendations related to ‘social status’ (82.4%) and ‘functional status/mobility’ (73.3%) were best adhered to. Recommendations related to ‘medication’ (53.2%) and ‘nutritional status’ (59.1%) were least adhered to. Adherence rates increased if recommendations were given to allied health professionals (OR = 6.37, 95% CI = 1.15–35.35) or by more experienced IGCTs (OR = 1.34, 95% CI = 1.04–1.72) and decreased when more recommendations were given (OR = 0.51, 95% CI = 0.33–0.80).

**Conclusion:**

Adherence rate to IGCT recommendations increased if given to allied health professionals or by more experienced IGCTs and when fewer recommendations were given. Study replication in an international multicenter study with a larger number of centers and evaluating the quality of the recommendations is suggested.

**Electronic supplementary material:**

The online version of this article (10.1007/s41999-020-00397-w) contains supplementary material, which is available to authorized users.

## Key summary points

**Aim:**

We evaluated the adherence to recommendations of inpatient geriatric consultation teams and the team and recommendation characteristics impacting the adherence rates.

**Findings:**

The overall adherence rate to the recommendations was 69.7%. Adherence rate increased if recommendations were given to allied health professionals or by more experienced consultation teams and when fewer recommendations were given.

**Message:**

Replication is needed in an international multicenter study with a specific attention given to the quality of the recommendations and the follow-up by the teams.

**Electronic supplementary material:**

The online version of this article (10.1007/s41999-020-00397-w) contains supplementary material, which is available to authorized users.

## Introduction

Due to demographic ageing, an increasing number of older people are being admitted to the hospital [1]. A majority of these older people suffer from multimorbidity, and functional, cognitive, and/or social needs that require specific attention [2, 3]. For these frail older patients, admission to an acute geriatric ward with adapted infrastructure and a multidisciplinary team results in a higher quality of care, decreased risk for hospital associated disability, fewer hospital readmissions and institutionalizations, and a higher degree of survival [4–6].

The limited capacity of geriatric wards has resulted in the implementation of alternative care models for frail older patients in the hospital [7]. One such model is the inpatient geriatric consultation team (IGCT), a mobile multidisciplinary team of geriatric experts who give recommendations based on a comprehensive geriatric assessment to the care teams of non-geriatric wards upon their request [8]. Several studies have demonstrated positive effects in terms of mortality, functional status, and cognition, but an overall consistent effect is lacking [4, 9, 10]. Nevertheless, the model has a high face validity and was implemented in several countries, because it is cheaper than expanding the capacity of geriatric wards [7, 11]. Researchers have argued that one of the main limitations hindering the full potential of this model of care is the gap between recommended and actual care [4, 12–14]. Indeed, reported adherence rates are often suboptimal with numbers ranging between 55.5% and 88% depending on the setting and population [13, 15–20]. Previous studies reported that less time between hospital admission and consultation, and limiting the number of recommendations to four were associated with higher adherence rates [13, 19]. Authors also hypothesized that the quality of the recommendations, the presence of multidisciplinary IGCT meetings, and systematic follow-up of recommendations impact the adherence rate [12, 13, 21].

Because multicenter evaluation studies of adherence to IGCT recommendations are lacking and the structure and team processes of IGCT in single-center studies are often poorly described, we set up a multicenter study specifically focusing on these crucial elements of recommendation-based IGCTs. Hence, the aims of this study were to describe the type and adherence rate of recommendations and to evaluate which team or recommendation characteristics influence the adherence to IGCT recommendations.

## Methods

### Design, sample, and setting

A multicenter observational study was conducted in ten hospitals in the Flemish speaking part of Belgium. A representative sample of IGCTs, based on geographical distribution and hospital type and size, was selected out of all IGCTs that had indicated interest to participate in the study.

Patients were eligible for participation in the study if they were older adults aged 75 years or older, Dutch-speaking, admitted to a non-geriatric ward, and evaluated by the IGCT. Data were collected for 30 consecutive patients in each hospital for whom an IGCT consult was requested by the non-geriatric ward. The study was approved by the Ethical Committee of the University Hospital Ghent, acting as central ethical committee (EC/2018/0097, EC/2018/0098), and the local ethical committees of all participating hospitals.

### Context

The structure and some of the processes of IGCTs are regulated in Belgium by means of a Royal Decree published in 2017 and revised in 2014 [22]. It states that each acute hospital with an acute geriatric ward has to organize a Care Program for Older Patients with the goal to improve the independency and quality of life of older patients with a geriatric risk profile through a multidisciplinary approach and guarantee continuity of care in the hospital and after discharge. Besides the acute geriatric ward, hospitals should have a geriatric day care hospital, an IGCT, and an external liaison service, and should offer consultations for outpatients by the geriatrician. The IGCTs should be an interdisciplinary team with minimum one geriatrician and 2–6 full-time equivalents of nurses and allied health care professionals, with the number of full-time equivalents financed by the government depending on the number of patients aged 75 years or older hospitalized on non-geriatric wards. The IGCT acts upon request of the physician of the non-geriatric ward in patients who are considered at risk based on screening with a validated instrument. The geriatrician can charge two honoraria per hospitalization for a bedside geriatric consult and two honoraria per week for a multidisciplinary team meeting.

### Variables

#### Patient characteristics

Data on age, gender, living situation, type of ward, and length of hospital stay of the included patients were collected.

#### IGCT composition and care processes

To collect data on the IGCT composition and care processes, a questionnaire was developed based on a literature review and expertise of the research team. The content validity of the questionnaire was measured in one round using the method of Lynn [23, 24]. Each question was evaluated by 11 experts in geriatric care for clarity (yes/no) and relevance (4-point Likert scale). A question was included in the questionnaire if it was rated as clear and ‘very relevant’ or ‘somewhat relevant’ by at least eight experts. Questions that were not considered clear were reformulated by the research team based on the suggestions given by the experts. Questions that were not considered relevant were removed, which was the case for three questions. The final questionnaire consisted of 26 questions, of which seven were related to hospital characteristics, two to the composition of the IGCT, and 17 to care processes of the IGCT.

The following variables were derived from the questionnaire and included in the multilevel model as potential predicators for adherence. Type of hospital was categorized as general versus university hospital. Type of ward was categorized as internal medicine versus surgical ward. Average experience of the IGCT was calculated by adding the number of years each IGCT member was working in the IGCT divided by the numbers of team members. The workload of the IGCT was calculated by dividing the total annual number of IGCT consultation request with the total number of full-time equivalents per team. Variables related to the IGCT structure were ‘participation in multidisciplinary team meetings on the non-geriatric wards (yes/no) and IGCT having a purely consultation role versus a co-management role.

#### IGCT recommendations

The IGCTs listed all the recommendations per included patient, indicated to which type of care professional that the recommendation was given and how it was communicated. The following variables were entered in the multilevel model as potential predictors: the average number of recommendations per patient, the way of communicating the recommendation (documented in record only, communicated face to face only, or both documented and communicated), the professional to whom the recommendation was given (medical doctor, nurse, allied health professional or non-geriatric team in case the recommendation was given to more than one professional group), and the number of care professionals receiving the intervention.

For each recommendation, the IGCT reported whether the recommendation was adhered to (i.e., completely adhered to, partially adhered to, not adhered to, do not know). The recommendations were thereafter grouped into eight thematic clusters, each with several subgroups, in an iterative process. The overall adherence rate was defined as the total number of recommendations that was completely adhered to over the total number of recommendations given. Partially adhered recommendations were considered not adhered to. Adherence rates per cluster were calculated in the same way.

### Data collection

Data were collected from March to June 2018. Prior to the start of the data collection, a research assistant (EV or EG) visited each hospital to explain the data collection procedures to the members of the IGCT. Each IGCT completed the questionnaire to map the hospital and team characteristics and filled out one checklist per patient detailing the patient characteristics and IGCT recommendations. Informed consent was obtained from all participants prior to data collection. After data collection, the anonymous patient checklists were collected by the research team.

### Data analysis

Descriptive analyses were conducted in SPSS version 25. Categorical variables were expressed as numbers and percentages, normally distributed continuous data as means and standard deviations, and non-normally distributed data as medians and interquartile ranges. Patients that had been assessed by the IGCT but where no recommendations were formulated (*n* = 5) were excluded from the analyses.

A multilevel logistic regression model was build using the stepwise nested model approach advocated by Hox and et al. for all recommendations for which adherence status was reported (*n* = 804) [23]. The IGCTs were defined as a random effect to account for the clustering of data. The potential predictors for adherence to the recommendations were defined as fixed effects. A limited number of predictors were selected by the research team to prevent overfitting the model because of the small number of centers (*k* = 10). First, an intercept only model was constructed to define the ‘null model’ as benchmark for the nested models. Second, predictors at the level of the recommendation were added to the intercept only model. Predictors with a *p* value > 0.2 were removed from the model using backward elimination. Third, the same approach was used for predictors at the level of the hospitals. Fourth, all predictors with a *p* value ≤ 0.2 at the level of the recommendation and the hospitals were combined in the final model. The ‘type of recommendation’ was forced into the model as a potential confounder for the other predictors. Odds ratios (OR) with 95% confidence intervals (CI) were constructed. At each step of the modelling strategy, the Akaike Information Criteria (AIC) were used to evaluate improvement in the ‘fit of the model’, and the likelihood ratio test was used to test the statistical significance of the improvement. A two-sided *p* value of < 0.05 was used to assess statistical significance. The predictive value of the fixed effect predictors was evaluated using the c-concordance statistic (C-Index) with 95% CI. The proportion of the variance explained (R2) was calculated using the ‘Snijders and Bosker’s’ approach [24].

## Results

### Description of the sample

Data of 292 patients were collected. Fourteen patients who were younger than 75 years or without primary outcome data were excluded from the analyses, resulting in an inclusion of 278 patients from 10 hospitals. Just over half of the sample were men and the mean age of the patients was 82.5 years (SD ± 5.0). Half of the patients was living at home with someone else, while 40.4% was living alone at home. The median length of hospital stay was 10 days (IQR 6–16). (See Table 1).

**Table 1:** Patient characteristics

Variable	*N* = 278
Age, mean (SD)	82.5 (5.0)
Male, *n* (%)	143 (51.4)
Living situation	
Living at home, alone	112 (40.4)
Living at home, together	139 (50.2)
Assisted living	9 (3.2)
Nursing home	17 (6.2)
Hospitalization ward, *n* (%)	
Internal medicine ward	129 (46.6)
Surgical ward	148 (53.4)
Length of hospital stay in days, median (IQR)	10 (6–16)
IGCT recommendations per patient, median (range)	3 (1–13)

*IGCT* inpatient geriatric consultation teams; *IQR* interquartile range; *SD* standard deviation

### Composition and modus operandi of IGCTs

The total number of full-time equivalents (FTE), excluding the geriatrician, ranged from 0.90 to 6.80 per IGCT (mean 3.4 ± SD 1.7). The nurse was a core member in all IGCTs. The dietician, physical therapist, speech therapist, social worker, and psychologist were available upon request in more than 50% of the teams (Table 2).

**Table 2:** Composition of consultation teams (*n* = 10)

Profession	Core member, *n* (%)	Available on request, *n* (%)
Nurse	10 (100)	0
Geriatrician	9 (90)	1 (10)
Occupational therapist	9 (90)	1 (10)
Psychologist	4 (40)	6 (60)
Dietician	3 (30)	7 (70)
Physical therapist	2 (20)	8 (80)
Speech therapist	2 (20)	8 (80)
Social worker	0	10 (100)

Multidisciplinary IGCT meetings were organized in all but one team with a frequency varying between one and five times per week. In five hospitals, members of the IGCT also participated in team meetings organized by the non-geriatric wards. Half of the teams systematically checked whether their recommendations had been adhered to. In case of non-adherence to the recommendations, eight teams indicated that they recontacted the person to whom the recommendation was given, seven also contacted other team members on the non-geriatric ward, and four contacted the treating physician. Four teams indicated to have a consultative role only, while six teams indicated to have an executive role for certain care interventions. Seven teams indicated to feel supported (excellent or good) by their division manager, while only three teams felt supported by the hospital management (Table 3).

**Table 3:** Hospital and team characteristics

Variable	*N* = 10
Type of hospital, *n* (%)	
General hospital	7 (70)
University hospital	3 (30)
Acute geriatric unit in the hospital	10 (100)
Geriatric daycare hospital	8 (80)
Year of IGCT establishment, *n* (%)	
2004	1 (10)
2007	5 (50)
2008	3 (30)
2014	1 (10)
Average years of experience in IGCT (excluding geriatrician), median (IQR)	11.0 (6.9–16.5)
Multidisciplinary IGCT meeting, *n* (%)	
Daily	2 (20)
3 ×/week	1 (10)
2 ×/week	2 (20)
1 ×/week	4 (40)
None	1 (10)
Participation at team meetings on non-geriatric wards, *n* (%)	5 (50)
Role of IGCT	
Consultation role	4 (40)
(Partly) executive (co-management) role	6 (60)
Follow-up of patients after initial assessment and recommendations, *n* (%)	
Never/rarely	0 (0)
Often	5 (50)
Always	5 (50)
Perceived support by division manager	
Excellent	2 (20)
Good	5 (50)
Sufficient	3 (30)
Perceived support by hospital management	
Excellent	1 (10)
Good	2 (20)
Sufficient	4 (40)
Insufficient	3 (30)

*IGCT* inpatient geriatric consultation teams; *IQR* interquartile range; *SD* standard deviation

### Adherence to IGCT recommendations

A total number of 942 recommendations was given with a median number of three recommendations (range 1–13) per patient. Recommendations related to ‘functional status/mobility’ (28.2%), ‘cognitive/mental status’ (17.0%), and ‘social status’ (16.9%) were most frequently given (see Table 4). Almost half of the recommendations (41.1%) were given by the IGCT nurse, 18.7% by the geriatrician, 13.6% by the occupational therapist, and 23% were recommendations given by the team. A very small number of recommendations were given by the social worker (*n* = 1), physical therapist (*n* = 2), or psychologist (*n* = 10). Recommendations were mainly communicated with nurses (59.1%), head nurses (28.5%), or physicians (51.5%), and less often with the occupational therapist (12.2%), social worker (11.6%), physical therapist (5.9%), dietician (3.4%), psychologist (1.3%), and speech therapist (0.5%) of the non-geriatric ward. About a quarter (26.6%) of the recommendations was also communicated to the general practitioner of the patient.

**Table 4:** Overview of the adherence rate to IGCT recommendations (*n* = 942)

Recommendation	Frequency *n* (%)	Adherence rate *n* (%)		
		Complete	Partial/no	Don’t know
Total number of recommendations	942	657 (69.7)	147 (15.6)	138 (14.6)
1. Nutritional status	98 (10.4)	58 (59.1)	25 (25.5)	15 (15.3)
Referral to dietician	31 (31.2)	20 (64.5)	7 (22.6)	4 (12.9)
Monitor food intake	20 (20.4)	13 (65.0)	3 (15.0)	4 (20.0)
Increase nutritional intake	2 (2.0)	2 (100)	0	0
Monitor weight	14 (14.3)	9 (64.3)	3 (21.4)	2 (14.3)
Dysphagia	23 (23.5)	8 (34.8)	12 (52.2)	3 (13.0)
Switch to specific diet	6 (6.1)	5 (83.3)	0	1 (16.7)
Miscellaneous	2 (2.0)	1 (50.0)	0	1 (50.0)
2. Cognitive/mental status	160 (17.0)	113 (70.6)	23 (14.4)	24 (15.0)
Delirium	49 (30.6)	37 (75.5)	9 (18.4)	3 (6.1)
Dementia	89 (55.6)	63 (70.8)	9 (10.1)	17 (19.1)
Sensory impairments	3 (1.9)	1 (33.3)	0	2 (66.7)
Depression	14 (8.8)	10 (71.4)	2 (14.3)	2 (14.3)
Miscellaneous	5 (2.5)	2 (40.0)	2 (60.0)	0
3. Medication	77 (8.2)	41 (53.2)	17 (22.1)	19 (24.7)
Start new drugs	48 (62.3)	20 (41.7)	12 (25.0)	16 (33.3)
Discontinue use of drugs	3 (3.9)	3 (100)	0	0
Medication review	21 (27.3)	15 (71.4)	4 (19.0)	2 (9.5)
Unspecified/other	5 (6.5)	3 (60.0)	1 (20.0)	1 (20.0)
4. Excretion/voiding	34 (3.6)	24 (70.6)	8 (23.5)	2 (5.9)
Bowel function	23 (67.6)	17 (73.9)	5 (21.7)	1 (4.3)
Bladder function	11 (32.4)	7 (63.6)	3 (27.3)	1 (9.1)
5. Functional status/mobility	266 (28.2)	195 (73.3)	39 (14.7)	32 (12.0)
Falls/fall risk	114 (42.9)	68 (59.6)	25 (21.9)	21 (18.4)
Start rehabilitation	65 (24.4)	52 (80.0)	7 (10.8)	6 (9.2)
Support ADL performance	52 (21.1)	45 (85.7)	5 (8.9)	2 (5.4)
Mobility	24 (9.0)	22 (91.7)	2 (8.3)	0
Support IADL performance	8 (1.5)	5 (62.5)	0	3 (37.5)
Frailty screening	3 (1.1)	3 (100)	0	0
6. Social status (living situation, discharge destination)	159 (16.9)	131 (82.4)	11 (6.9)	17 (10.7)
Referral to social worker	65 (40.9)	53	7	5
Organize alternative to living at home	8 (5.0)	7 (87.5)	1 (12.5)	0
Transfer to rehabilitation center	13 (8.2)	13 (100)	0	0
Transfer to geriatrics department	11 (6.9)	11 (100)	0	0
Expanding existing professional home care	57 (35.8)	44 (77.2)	2 (3.5)	11 (19.3)
Miscellaneous	5 (3.1)	3 (60.0)	1 (20.0)	1 (20.0)
7. Medical	86 (7.1)	55 (64.0)	11 (12.8)	20 (23.3)
Monitor fluid/electrolyte balance	4 (4.7)	1 (25.0)	3 (75.0)	0
Additional tests and investigations	18 (20.9)	14 (77.8)	2 (11.1)	2 (11.1)
Adequate oxygen delivery	3 (3.5)	3 (100)	0	0
Referral to other specialists	49 (57.0)	29 (59.2)	4 (8.2)	16 (32.7)
Miscellaneous	12 (14.0)	8 (66.7)	2 (16.7)	2 (16.7)
8. Other	62 (6.6)	40 (64.5)	13 (21.0)	9 (14.5)
Pain	18 (29.0)	13 (72.2)	2 (11.1)	3 (16.7)
Palliative care	9 (14.5)	8 (88.9)	1 (11.1)	0
Prevention of pressure ulcers	34 (54.9)	18 (52.9)	10 (55.6)	6 (17.6)
Miscellaneous	1 (1.6)	1 (100)	0	0

*ADL* activities of daily living; *IADL* instrumental activities of daily living

The recommendations of each patient were communicated in different ways to the non-geriatric care team. The majority of the recommendations were communicated in person (67.6%), registered in the electronic patient file (64.8%), or entered in a separate IGCT report (55.8%). Recommendations were less often communicated by e-mail (24.1%), by phone (18.3%), or written in a paper-based patient file (19.2%).

The overall adherence to the IGCT recommendations was 69.7% and varied between hospitals from 54 to 100%. At the cluster level, recommendations regarding ‘social status’ (82.4%) and ‘functional status/mobility’ (73.3%) were best adhered to while recommendations regarding ‘medication’ (53.2%) and ‘nutritional status (59.1%) were the least often adhered to (see Table 4).

### Factors influencing the adherence rate of IGCT recommendations

The multilevel logistic regression resulted in a final model with six predictors (See Table 5). A statistically significant increase in adherence was observed if the IGCT recommendation was given to allied health professionals (OR = 6.37, 95% CI = 1.15–35.35) and if the IGCT was more experienced (OR = 1.34, 95% CI = 1.04–1.72). The adherence rate increased when fewer recommendations were given (OR = 0.51, 95% CI = 0.33–0.80). An association was observed for the predictor’s number of health professionals receiving the recommendation, participation during multidisciplinary team meetings, and having a co-management role. However, the estimates were not precise enough to infer statistical significance.

**Table 5:** Predictors of higher adherence rate to IGCT recommendations

Predictors in final model	OR (95% CI)	*p* value
Recommendation given to		
Medical doctor	1.87 (0.36–9.69)	0.456
Nurse	1.34 (0.29–6.25)	0.714
Allied health professional	6.37 (1.15–35.35)	0.034
Team	1.23 (0.15–10.24)	0.846
Number of care professionals receiving the recommendation	1.91 (0.94–3.86)	0.073
Average experience (years) of IGCT	1.34 (1.04–1.72)	0.025
Participation in multidisciplinary team meeting	3.19 (0.66–15.43)	0.148
Average number of recommendations given	0.51 (0.33–0.80)	0.003
Co-management role (versus consultation role)	3.08 (0.81–11.67)	0.099

Predictors omitted from final model	OR (95% CI)	*p* value
Recommendation communicated through		
Documentation in patient record	0.88 (0.49–1.59)	0.672
Personal contact	0.96 (0.41–2.27)	0.930
Number of sources in which the recommendation was documented	0.90 (0.60–1.36)	0.622
Workload IGCT	1.00 (0.99–1.01)	0.662
Recommendation given in university hospital	0.78 (0.36–1.69)	0.522
Recommendation given in internal medicine ward	1.28 (0.85–1.92)	0.240

Summary of the final model: 10 clusters with 804 observations. ICC = 0.14. AIC intercept only model = 705; AIC level 1 predictors only = 688; AIC level 2 predictors only = 695; AIC final model = 686. R2 level 1 = 0.08; R2 level 2 = 0.34. Standard deviation of random effect parameter = 0.74 (95% CI 0.38–1.45)

*OR* odds ratio; *IGCT* inpatient geriatric consultation team; *CI* confidence interval

The predictors discriminated good between adherence and non-adherence (C-index = 0.79, 95% CI = 0.75–0.83). Predictors at the level of the IGCTs were better in explaining the adherence to the recommendations (*R*2 = 0.34), than predictors at the level of the recommendations (*R*2 = 0.08). The final two-level model had a better ‘model fit’ demonstrating that the adherence was best explained in the model with predictors at both levels (*p* = 0.002).

## Discussion

In this multicenter study including ten IGCTs being operational for several years, a mean adherence rate of 69.7% to the IGCT recommendations was observed. This is higher than the 56.8% adherence rate reported in a single-center study conducted in 2010 in older patients undergoing hip surgery in one of the university hospitals included in this sample, but was lower than the 78% reported by Morin et al. [13]. This can likely be explained by differences in the design. Morin et al. [13] phoned all included patients 3 months after hospital discharge to map the adherence rate to recommendations concerning the postdischarge situation (i.e., follow-up by a general practitioner, starting additional home care support). In our study, there were no explicit instructions to contact GPs or patients postdischarge to check whether recommendations related to the postdischarge period were implemented. This has resulted in a higher number of recommendations of which the adherence rate was unknown and, consequently, a lower observed complete adherence rate.

We observed a higher adherence rate if IGCT recommendations were given to allied health professionals, such as social workers, occupational therapists, physical therapists, or dieticians. We assume that this is due to the fact that these interventions can be directly implemented. This is in contrast with more complex interventions, such as exploring the underlying factors of a delirium, which require more extensive geriatric knowledge and expertise. Indeed, health care professionals on non-geriatric wards often lack knowledge and expertise regarding geriatric care as they have less exposure, but also because geriatric care aspects are still insufficiently addressed in both nursing and medical curricula [25–27]. This suggests that breaking down complex interventions into simple direct interventions would improve adherence rates. Our analyses also demonstrated that more experienced IGCTs reach higher adherence rates. This can be linked to the previous argumentation, but might also be explained by the credibility of the consultants towards their colleagues, team dynamics, positive past experiences, and appropriate communication skills that increase the likelihood of adherence. Our findings suggest that successfully taking up the role of consultant generally takes time and that teams should, therefore, ideally be a mix of both junior and senior consultants to safeguard the continuity and available expertise in the team over time.

Limiting the number of IGCT recommendations had been proven successful in improving the adherence rates by Morin et al. [13] and these findings were confirmed in our study. Listing too many recommendations presumably makes it more difficult for the consultees to prioritize the suggested care interventions, again likely due to lack of knowledge to determine which recommendations are more likely to improve patient outcomes. Limiting and prioritizing recommendations should, therefore, be advised to all IGCTs.

Although not statistically significant, probably due to the limited number of clusters and power in the study, our findings indicated that IGCTs that communicate their recommendations to more individuals and are present at the multidisciplinary team meeting on the non-geriatric wards are more likely to get their recommendations translated into actual care interventions. These are strategies that can both increase knowledge and awareness of geriatric care, because it are opportunities to explain face to face why certain interventions are recommended, what they entail and how urgent they are [28].

Finally, teams that reported having at least partly a co-management role also saw their recommendations better adhered to. Moving from a consultation model to a co-management model has long been suggested to overcome the gap between recommendation and intervention [29–31] and is also reflected in a recent european survey where one in three hospitals indicated plans to implement geriatric co-management within the next 5 years [7]. Geriatric co-management has also been recommended as the favoured care model in the 2018 consensus statement for the management of frail patients undergoing surgery [32]. However, legislative norms or national care programmes will need to be developed in countries where these are not yet present as these have been instrumental in the successful implementation of both consultative and co-management services [7].

Several methodological elements should be considered when interpreting the results. It is likely that teams with a perceived higher adherence rate were more likely to participate in the study and that the reported adherence rates are, therefore, not generalizable to all IGCTs. Also the fact that data were collected through self-reporting might have resulted in reporting bias overestimating the actual adherence. Data collection through observation by an independent researcher strengthens the reliability and quality of the data, but would have been too time and resource intensive. We also did not evaluate the quality of the recommendations given and one can question which care interventions would have been done regardless of the IGCT consult. We did not measure the frequency of follow-up visits by the IGCT at patient level. As follow-up is an essential part of CGA, future studies should include this variable in their analysis. The number of clusters (i.e., ten hospitals) is considered a minimum to conduct multilevel analyses and explains why the estimates of the significant predictors were not precise and why type II errors are probable for the nonsignificant predictors. Nevertheless, we observed a significant variance in adherence rates between the teams allowing us to study which team and recommendation characteristics are associated with higher adherence rates in the first multicenter study.

In conclusion, the overall adherence rate to IGCT recommendations was 69.7% and was the highest for recommendations related to ‘social status’ and ‘functional status/mobility’. Adherence rates increased if recommendations were given to allied health professionals or by more experienced IGCTs and when fewer recommendations were given. Replication of the study in an international multicenter study with a larger number of clusters that also evaluates the quality of the care recommendations and follow-up by the IGCT is suggested.

## Electronic supplementary material

Below is the link to the electronic supplementary material.

Supplementary file1 (DOCX 19 kb)

## Data Availability

The anonymous data set is available upon reasonable request to the corresponding author.
