# Supplementary material for: Adherence to recommendations of inpatient geriatric consultation teams: a multicenter observational study
Source: Eur Geriatr Med. 2020 Sep 25;12(1):175–84. doi: 10.1007/s41999-020-00397-w (PMC7900061; doi:10.1007/s41999-020-00397-w)
Supplement: Supplementary file 1 — Supplementary file1 (DOCX 19 kb) [file 41999_2020_397_MOESM1_ESM.docx]

***Extended versions Table 4:*** *Overview of the adherence rate to IGCT recommendations (n = 942)*

| **Recommendation** | **Frequency n (%)** | **Adherence rate**  **n (%)** | | |
| --- | --- | --- | --- | --- |
|  |  | **Complete** | **Partial/no** | **Don’t know** |
| **Total number of recommendations** | **942** | **657 (69.7)** | **147 (15.6)** | **138 (14.6)** |
| 1. **Nutritional status** | **98 (10.4)** | **58 (59.1)** | **25 (25.5)** | **15 (15.3)** |
| - 1. Referral to dietician | 31 (31.2) | 20 (64.5) | 7 (22.6) | 4 (12.9) |
| - 1. Monitor food intake | 20 (20.4) | 13 (65.0) | 3 (15.0) | 4 (20.0) |
| - 1. Increase nutritional intake | 2 (2.0) | 2 (100) | 0 | 0 |
| - 1. Monitor weight | 14 (14.3) | 9 (64.3) | 3 (21.4) | 2 (14.3) |
| - 1. Dysphagia | 23 (23.5) | 8 (34.8) | 12 (52.2) | 3 (13.0) |
| - *Referral to language therapist* | *21* | *7* | *11* | *3* |
| - *Preventive measures for safe swallow act* | *2* | *1* | *1* | *0* |
| - 1. Switch to specific diet | 6 (6.1) | 5 (83.3) | 0 | 1 (16.7) |
| - 1. Miscellaneous | 2 (2.0) | 1 (50.0) | 0 | 1 (50.0) |
| 1. **Cognitive / mental status** | **160 (17.0)** | **113 (70.6)** | **23 (14.4)** | **24 (15.0)** |
| - 1. Delirium | 49 (30.6) | 37 (75.5) | 9 (18.4) | 3 (6.1) |
| - *Prevention* | *40* | *32* | *5* | *3* |
| - *Pharmacological treatment of hyperactive delirium* | *2* | *0* | *2* | *0* |
| - *Search for underlying cause* | *2* | *0* | *2* | *0* |
| - *Unspecified* | *5* | *5* | *0* | *0* |
| - 1. Dementia | 89 (55.6) | 63 (70.8) | 9 (10.1) | 17 (19.1) |
| - *Referral for additional testing at geriatric day clinic* | *18* | *11* | *2* | *5* |
| - *Inhospital testing (e.g. MMSE, clock-drawing test)* | *64* | *52* | *6* | *6* |
| - *Follow up on mental status (unspecified)* | *7* | *0* | *1* | *6* |
| - 1. Sensory impairments | 3 (1.9) | 1 (33.3) | 0 | 2 (66.7) |
| - 1. Depression | 14 (8.8) | 10 (71.4) | 2 (14.3) | 2 (14.3) |
| - 1. Miscellaneous | 5 (2.5) | 2 (40.0) | 3 (60.0) | 0 |
| 1. **Medication** | **77 (8.2)** | **41 (53.2)** | **17 (22.1)** | **19 (24.7)** |
| - 1. Start new drugs using START criteria | 48 (62.3) | 20 (41.7) | 12 (25.0) | 16 (33.3) |
| - 1. Discontinue use of drugs using STOPP criteria | 3 (3.9) | 3 (100) | 0 | 0 |
| - 1. Medication review | 21 (27.3) | 15 (71.4) | 4 (19.0) | 2 (9.5) |
| - 1. Unspecified/other | 5 (6.5) | 3 (60.0) | 1 (20.0) | 1 (20.0) |
| 1. **Excretion/voiding** | **34 (3.6)** | **24 (70.6)** | **8 (23.5)** | **2 (5.9)** |
| - 1. Bowel function | 23 (67.6) | 17 (73.9) | 5 (21.7) | 1 (4.3) |
| - *Monitor voiding pattern* | *19* | *13* | *5* | *1* |
| - *Treatment of constipation* | *4* | *4* | *0* | *0* |
| - 1. Bladder function | 11 (32.4) | 7 (63.6) | 3 (27.3) | 1 (9.1) |
| - *Remove urinary catheter* | *4* | *3* | *1* | *0* |
| - *Bladder scan* | *5* | *3* | *2* | *0* |
| - *Other* | *2* | *1* | *0* | *1* |
| 1. **Functional status / mobility** | **266 (28.2)** | **195 (73.3)** | **39 (14.7)** | **32 (12.0)** |
| - 1. Falls / fall risk | 114 (42.9) | 68 (59.6) | 25 (21.9) | 21 (18.4) |
| - *Referal to fall clinic* | *10* | *8* | *1* | *1* |
| - *Safe footwear* | *6* | *2* | *2* | *2* |
| - *Screening for osteoporosis* | *2* | *2* | *0* | *0* |
| - *Orthostatism* | *39* | *20* | *14* | *5* |
| - *Patient information on fall prevention* | *49* | *29* | *7* | *13* |
| - *Screening for fall risk* | *8* | *7* | *1* | *0* |
| - 1. Start rehabilitation | 65 (24.4) | 52 (80.0) | 7 (10.8) | 6 (9.2) |
| - *Referral to physical therapist* | *33* | *25* | *5* | *3* |
| - *Referral to occupational therapist* | *2* | *2* | *0* | *0* |
| - *Unspecified* | *30* | *25* | *2* | *3* |
| - 1. Support ADL performance | 52 (21.1) | 45 (85.7) | 5 (8.9) | 2 (5.4) |
| - *Provide assistance with bathing, dressing, toileting* | *9* | *6* | *2* | *1* |
| - *Referal to occupational therapist* | *29* | *25* | *3* | *1* |
| - *Unspecified* | *14* | *14* | *0* | *0* |
| - 1. Mobility | 24 (9.0) | 22 (91.7) | 2 (8.3) | 0 |
| - *Recommending / providing walking aid* | *21* | *19* | *2* | *0* |
| - *Miscellaneous* | *3* | *3* | *0* | *0* |
| - 1. Support IADL performance | 8 (1.5) | 5 (62.5) | 0 | 3 (37.5) |
| - 1. Frailty screening | 3 (1.1) | 3 (100) | 0 | 0 |
| 1. **Social status (living situation, discharge destination)** | **159 (16.9)** | **131 (82.4)** | **11 (6.9)** | **17 (10.7)** |
| - 1. Referral to social worker | 65 (40.9) | 53 | 7 | 5 |
| - 1. Organizing (temporary) alternative to living at home | 8 (5.0) | 7 (87.5) | 1 (12.5) | 0 |
| - 1. Organizing transfer to rehabilitation center | 13 (8.2) | 13 (100) | 0 | 0 |
| - 1. Organizing transfer to geriatrics department | 11 (6.9) | 11 (100) | 0 | 0 |
| - 1. Expanding existing professional home care | 57 (35.8) | 44 (77.2) | 2 (3.5) | 11 (19.3) |
| - 1. Miscellaneous | 5 (3.1) | 3 (60.0) | 1 (20.0) | 1 (20.0) |
| 1. **Medical** | **86 (7.1)** | **55 (64.0)** | **11 (12.8)** | **20 (23.3)** |
| - 1. Monitor fluid and electrolyte balance | 4 (4.7) | 1 (25.0) | 3 (75.0) | 0 |
| - 1. Additional tests and investigations | 18 (20.9) | 14 (77.8) | 2 (11.1) | 2(11.1) |
| - 1. Adequate oxygen delivery | 3 (3.5) | 3 (100) | 0 | 0 |
| - 1. Referral to other specialists | 49 (57.0) | 29 (59.2) | 4 (8.2) | 16 (32.7) |
| - 1. Miscellaneous | 12 (14.0) | 8 (66.7) | 2 (16.7) | 2 (16.7) |
| 1. **Other** | **62 (6.6)** | **40 (64.5)** | **13 (21.0)** | **9 (14.5)** |
| - 1. Pain | 18 (29.0) | 13 (72.2) | 2 (11.1) | 3 (16.7) |
| - *Monitor pain* | *10* | *8* | *0* | *2* |
| - *Pain treatment* | *6* | *4* | *2* | *0* |
| - *Referral to pain nurse consultant* | *2* | 1 | 0 | 1 |
| - 1. Palliative care | 9 (14.5) | 8 (88.9) | 1 (11.1) | 0 |
| - *Referral to palliative support team* | *6* | *6* | *0* | *0* |
| - *Miscellaneous* | *3* | *2* | *1* | *0* |
| - 1. Prevention of pressure ulcers | 34 (54.9) | 18 (52.9) | 10 (55.6) | 6 (17.6) |
| - 1. Miscellaneous | 1 (1.6) | 1 (100) | 0 | 0 |

*ADL = Activities of Daily Living ; IADL = Instrumental Activities of Daily Living; MMSE = Mini-Mental State Examination;*
